# Autotoxicity of Diterpenes Present in Leaves of *Cistus ladanifer* L.

**DOI:** 10.3390/plants8020027

**Published:** 2019-01-22

**Authors:** Natividad Chaves Lobón, Irene Ferrer de la Cruz, Juan Carlos Alías Gallego

**Affiliations:** Department of Plant Biology, Ecology and Earth Sciences, Faculty of Science, University of Extremadura, 06006 Badajoz, Spain; iferrerd@alumnos.unex.es (I.F.d.l.C.); jalias@unex.es (J.C.A.G.)

**Keywords:** autoallelopathy, terpenes, phenols

## Abstract

*Cistus ladanifer* has been described as an allelopathic and autoallelopathic species, and the compounds that could be involved in its autotoxicity are the flavonoids and diterpenes present in the exudate of its leaves. The aim of this study was to determine which family of compounds, either phenols or terpenes, are responsible for the autoallelopathic activity quantified in *C. ladanifer*. These compounds were extracted from the exudate of young leaves collected in spring and separated by column chromatography into two fractions: diterpenes and flavonoids. The obtained results showed that flavonoids, at the tested concentrations, did not have a negative effect on any of the parameters quantified in the germination process of *C. ladanifer* seeds. On the other hand, the germination, seedling size and seedling establishment, quantified through the germination index and rate, were negatively affected by the tested diterpene solutions. In view of the obtained results, it was concluded that the compounds involved in the autoallelopathy process of *C. ladanifer* are diterpenes.

## 1. Introduction

Allelopathy is a negative interaction by which secondary metabolites, produced by plants, harm a different receiver plant, granting the donor plant the advantage to compete for limited resources [1,2]. When the receiver plant is also the donor, the phenomenon is called autoallelopathy or autotoxicity, which is a type of intraspecific allelopathy [3,4]. Autotoxicity is often thought to cause replant problems [5,6], is common in woodlands, and is one of the major reasons for growth reduction under continuous monoculture. Autotoxicity plays an important role in natural and managed forest ecosystems, often causing problems in natural or artificial regeneration [7,8,9].

*Cistus ladanifer* (rock-rose) is an invasive species, which colonizes highly degraded soils and lives under extreme conditions and low fertility, as a result of the development of different adaptive mechanisms [10]. This species, through its leaves and stems, secretes an exudate that is rich in secondary metabolites [11]. The synthesis of these compounds could be a defense mechanism against stress [12], protecting the plant from ultraviolet radiation, high temperatures, and herbivores, and preventing competition with other species, as these compounds also have allelopathic effects [13,14,15].

The main compound families of the exudate of this species are phenols (aglycone flavonoids such as: Apigenin, 4′-methyl-apigenin, 7-methyl-apigenin, 7,4′-dimethyl-apigenin, 3-methyl-kaempferol, 3,4-dimethyl-kaempferol, 3,7-dimethyl-kaempferol, and 3,7,4′-trimethyl-kaempferol; [15,16]) and terpenes (monoterpenes, such as borneol, camphor, 3-carene, carvacrol, carvone, citral, 1,8-cineole, citronelol, limonene, myrtenal, nerol, pinene, piperitone, pulegone, terpinen-4-ol and thymol; and diterpenes such as: oxocativic acid, labdanolic acid, 7-oxo-8-labden-15-oic acid, and 6-acetoxi-7-oxo-8-labden-15-oic acid; [15,17,18,19]). Previous studies have reported the inhibitory and retardant effect of flavonoids, including apigenin; 4´-O-methylapigenin; 7-O-methylapigenin; 3-O-methylkaempherol; 3,7 di-O-methylkaempherol, and diterpenes, such as oxocativic acid, 7-oxo-8-labden-15-oic acid, and 6-acetoxi-7-oxo-8-labden-15-oic acid, on germination of herbaceous species such as *Rumex crispus*, *Triticum* sp. and *Latuca sativa* [19,20,21]. These studies demonstrate their phytotoxic activity, and that higher or lower activity of these compounds depends on the target species. 

Field studies have reported that the colonization of space next to an area occupied by a population of rock-rose is fast, likely due to the large number of seeds that this species produces [22]. However, in the center of a developed population of rock-rose, the establishment of new plants is very low and few studies explain the reasons for this behavior [23]. One of these reasons could be that this species may be autoallelopathic. Studies with soils from rock-rose populations, where diterpenes and flavonoids from the exudate of these plants were quantified [24], showed that these soils inhibited the germination of seeds and the growth of seedlings of *C. ladanifer*. These results demonstrated that this shrub could be an autotoxic species, showing the possible involvement of flavonoids and diterpenes in autotoxicity and the regeneration of the rock-rose population [24].

The compounds characterized in the exudate of *C. ladanifer* (flavonoids and diterpenes), however, differ in several respects:

- Both show seasonal variation in their synthesis, but at different seasons. The flavonoids of the exudate vary from five or six (winter) to 22–25 (summer) mg flavonoids/g of leaves [12], increasing three or four folds during the summer. Diterpenes show their maximum and minimum concentration in winter (20.93 mg/g dry-w) and summer (8.57 mg/g dry-w), respectively [19].
- Temperature is a determining factor in the synthesis of these compounds, and its effects also differ between the two families. High temperatures influence the qualitative composition of the secretion of flavonoids [12,13], and low temperature is the determining factor in the synthesis of diterpenes [19].
- Flavonoids and diterpenes of *C. ladanifer* have been quantified in the soil [24] and their incorporation route differs depending on the kind of compound. Diterpenes are incorporated mainly by leaf leaching and litter, whereas flavonoids are included in the soil through litter degradation [25].

Given these differences, the involvement of flavonoids and diterpenes in the autotoxicity of *C. ladanifer* could be different. Therefore, the aim of this work was to establish which compound family, phenols and/or terpenes, are involved in the autotoxic activity of *C. ladanifer*. 

## 2. Results

### 2.1. Germination Percentage

**Figure 1:**
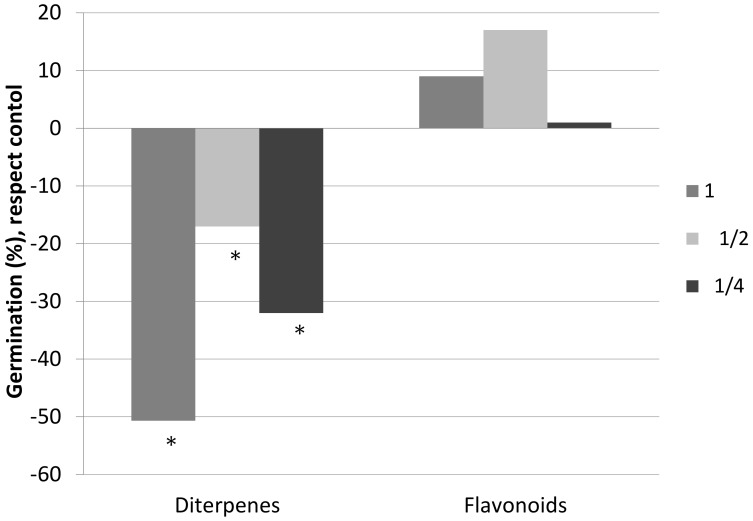
Germination percentage, with respect to the control sample, of the seeds of *C. ladanifer* watered with a solution of diterpenes and a solution of flavonoids at three different concentrations (6 replicates per treatment): 1 (0.26 g/L), ½ (0.13 g/L), and ¼ (0.065 g/L). * Significant difference with respect to the control sample (M-W; *p* < 0.05).

Figure 1 shows the effect of the diterpenes and flavonoids present in the exudate of *C. ladanifer* on the germination of its own seeds.

These tests show a clear negative effect of diterpenes on the germination of *C. ladanifer*, with a significant inhibition exerted by all three concentrations. On the other hand, when the seeds of *C. ladanifer* were watered with solutions containing flavonoids, no negative effects were observed on the germination of the seeds. It is worth highlighting that the 1 and ½ concentrations of flavonoids showed positive stimulation, although it was not significantly different from that obtained from the control. 

### 2.2. Germination Rate

When the germination rate (average germination time that needed) of the seeds of *C. ladanifer,* treated with the two solutions, was quantified (Table 1), the negative effect of diterpenes on this parameter was also observed, since germination was delayed when all three concentrations were used. The most concentrated solution caused the longest delay in germination time, with nine days needed for the seeds to germinate compared to the four days required by the control. Although there were no significant differences between the concentrations, the delay was shorter in more diluted concentrations, ranging from nine to seven days. 

As in the case of germination percentage, watering the seeds with the solutions that contained flavonoids did not significantly affect the germination rate, as no significant differences were observed between the tested concentrations. 

### 2.3. Germination Rate Index

The germination rate index is the average number of seeds that germinate per day and, once again, it is diterpenes which negatively affected this parameter. The results show that the value obtained is significantly lower in samples with the three concentrations of diterpenes tested (Table 2). Moreover, there was a concentration-dependent effect, which was significantly lower at the maximum concentration. 

The tests with the solutions containing flavonoids, as in the case of the other quantified parameters, did not significantly affect the number of germinated seeds per day, at any of the tested concentrations. It is worth highlighting that a slight stimulation was observed at concentration ½, although the difference was not significant with respect to the control, or any of the other flavonoid concentrations. 

### 2.4. Development of the Seedlings of C. ladanifer

**Figure 2:**
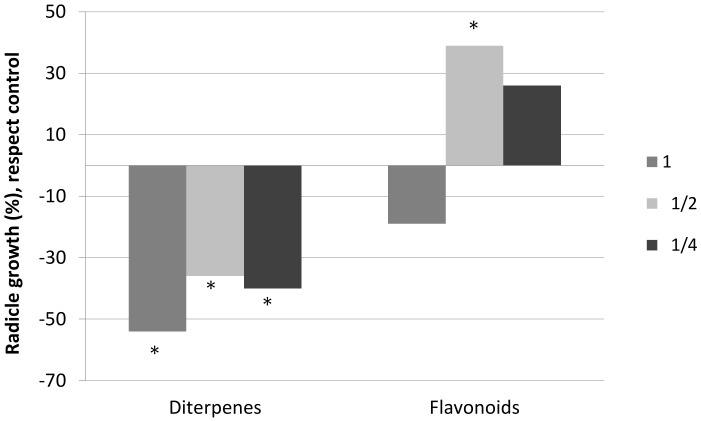
Radicle growth, with respect to the control, of the seedlings of *C. ladanifer* watered with solutions of diterpenes and solutions of flavonoids at three different concentrations: 1 (0.26 g/L), ½ (0.13 g/L), and ¼ (0.065 g/L). * Significant difference with respect to the control (M-W; *p* < 0.05).

**Figure 3:**
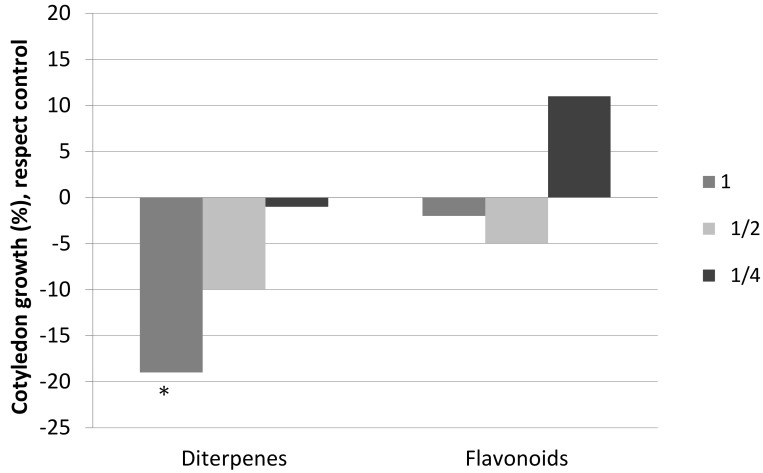
Cotyledon growth, with respect to the control, of the seedlings of *C. ladanifer* watered with solutions of diterpenes and solutions of flavonoids at three different concentrations: 1 (0.26 g/L), ½ (0.13 g/L), and ¼ (0.065 g/L). * Significant difference with respect to the control (M-W; *p* < 0.05).

The obtained results of the effects of diterpenes and flavonoids on the development of the roots and cotyledons of the seedlings of *C. ladanifer* differ, once again, in the compounds tested (Figure 2 and Figure 3). Diterpenes significantly inhibited the size of the roots and cotyledons, although there were differences in the effects on these parameters. The negative effect on the roots was considerably greater than the effect on the cotyledons, with the negative effect on the roots being similar for the three concentrations tested. The negative effect on the cotyledons disappeared at concentration ½.

On the other hand, flavonoids did not inhibit the size of the roots and cotyledons significantly, and they even provoked stimulation at low concentrations. As can be observed in Figure 2 and Figure 3, root size was significantly higher at concentration ½ with respect to the control concentration. At concentration ¼, there was a stimulation of the sizes of both cotyledons and roots, although it was not significant.

## 3. Discussion

In plants, the production and accumulation of secondary metabolites that act as allelochemicals and inhibit the germination and growth of other species is an adaptive strategy to increase the chances of survival of the producing plant, however, the result of this interaction goes beyond the effect caused between the two species. For example, the results obtained by Herranz et al. [26] suggest that the allelopathic effect of *C. ladanifer* may influence the composition and structure of the Mediterranean communities where this species is present, since it hinders the establishment of some sub-climatic species. 

Apart from affecting the establishment of coexisting species, allelopathic species can also affect their own establishment and self-regeneration. This is the case for *C. ladanifer*, which inhibits the germination and growth of species that share its habitat, and has also proved to be autotoxic, as has been demonstrated in studies conducted by Alías et al. [24]. 

The results obtained in this work show that the effects of the compounds that make up the exudate of *C. ladanifer* depend on the compound family. When the seeds of *C. ladanifer* were watered with the solution containing flavonoids, there was no negative effect on any of the quantified parameters at any of the concentrations tested. Taking into account that phenols are a compound group associated with the toxicity of some species classified as allelopathic [27], these results could be considered to be in disagreement. In fact, Sosa et al. [28] showed the toxicity of some of the flavonoids, present in the exudate of *C. ladanifer*, on the germination and growth of some herbaceous species (both separately and in combination). This difference in the activity of flavonoids may be due to the fact that the degree of toxic action of these compounds depends on the target species, as has also been demonstrated in previous studies [29,30]. It is worth highlighting that flavonoids would not only be involved in the autotoxicity of *C. ladanaifer*, as low concentrations seemed even to stimulate germination, cotyledon size and root size, with the latter being significantly stimulated. It is already known that some compounds act as plant growth regulators, exhibiting hormesis, or concentration-dependent stimulatory or inhibitory effects on seedling growth [31,32]. Weir et al. [9] discovered that (−)-catechin isolated from *Centaurea maculosa* stimulated root growth in *Gaillardia aristata* and *Lobelia erinus* at 10 μg/mL, but had a significant inhibitory effect at 400 μg/mL. Studies conducted with 3,4-dihydroxyacetophenone had similar effects on some of the plants tested in this study, showing increased growth of radish shoot and rice root at lower concentrations of this compound [33]. The fraction of flavonoids with which the study was carried out could have these characteristics.

When the seeds of *C. ladanifer* were watered with the solution containing diterpenes, the germination, germination rate and the root size of the seedlings were significantly inhibited, even at the lowest concentration tested. Cotyledon size was negatively affected only at the highest concentration tested. Diterpenes not only inhibited germination, but also the germination rate, which is a parameter that provides a closer and more realistic view of the activity of these molecules as possible allelopathic agents in the natural medium, and their potential use as natural herbicides [34]. The delay in the germination rate is very important for the establishment of a species under natural conditions, since it affects the chance of a plant to establish, as it cannot make use of favorable conditions to do so. On the other hand, the negative effect on root size was higher than that produced on cotyledon size and germination itself, which has been observed in other studies, for both terpenes and phenols [35,36]. This can be attributed to the fact that roots are the first to sense and respond to allelochemicals from the environment, and are particularly sensitive to allelochemicals [37,38].

It is worth highlighting that the effectiveness of diterpenes as autotoxic compounds, at the tested concentrations, is similar to that quantified for other autotoxic compounds, such as b-cembrenediol, di-n-hexyl phthalate, ergosta-5-en-3-ol, 1-tetradecene, and octacosane in flue-cured tobacco [3], as well as 3,4-dihydroxyacetophenone in *Picea schrenkiana* [33], trans-cinnamic acid, p-coumaric acid, and ferulic acid in *Panax quinquefolium* (7). 

The synthesis of flavonoids and diterpenes in the leaves of *C. ladanifer* is clearly season-dependent [12,19], with maximum production of flavonoids and diterpenes in summer and autumn-winter, respectively. This seasonal variability is determined by the environmental factors that induce the synthesis of these compounds; water stress and ultraviolet light are the main inducing factors for the production of flavonoids, and low temperatures induce the production of diterpenes [12,19]. These differences in the factors that determine the synthesis of these two compound groups may lead to thinking that the roles they play in the plant could be different. For example, although allelopathic activity has been attributed to both flavonoids and diterpenes, the target species on which they act may be different. Flavonoids may act more negatively on herbaceous species, which are predominant in spring, and diterpenes could inhibit the germination of the producing species, which takes place in late autumn and early winter [22]. This hypothesis gains more strength when considering the way in which these compounds enter the soil [25]. Flavonoids are mainly incorporated into the soil through the decomposition of dead leaves (which fall mostly at the end of spring) and diterpenes do so through the bleaching caused by the rain. The rainy period in rock-rose ecosystems is during autumn-winter, which is, in turn, when the germination of the seeds of *C. ladanifer* takes place and when diterpenes would enter the soil. 

In conclusion, the results obtained in this study show that, of the compounds present in the exudate of *C. ladanifer*, diterpenes inhibited the germination and growth of *C. ladanifer* seedlings. Therefore, diterpenes compounds would be the ones involved in its autotoxicity. Further studies are required to determine the concentration of these compounds in *C. ladanifer* soil and the stability of those compounds in the soil. 

## 4. Materials and Methods 

### 4.1. Sample Collection

The place selected for the collection of samples was a rock-rose population located in Olivenza (38^0^ 46′ 21″ N-7^0^ 10′ 14.66″ W). Samples of leaves and seeds were collected from several individuals that were randomly selected. The fruits were collected in august (once they had matured in the plant) and then stored in darkness until the beginning of the study. Fruits are woody capsules with eight to ten locules (carpels) containing seeds approx. 0·8 × 0·6 mm in size. The leaves were collected in spring. The types of leaf selected were shoots that appeared in that season and leaves born during the previous autumn. 

### 4.2. Extraction of Flavonoids and Diterpenes

For the complete extraction of compounds present in the exudate, 200 g of leaves of *C. ladanifer* were placed in 400 mL of chloroform [24]. The content was shaken for one hour to facilitate the extraction of the allelopathic compounds, and then it was filtered using 14 mm filter paper. The chloroform was evaporated at room temperature and the resulting extract was resuspended in methanol. This solution was frozen at –20 °C for 12 h to allow the wax to precipitate, which was removed by centrifuge at 10,000 rpm [39]. 

### 4.3. Chromatographic Separation

For the purification of flavonoids and diterpenes from the leaves of *C. ladanifer,* the exudate was previously separated into several fractions by column chromatography at room temperature. To this end, the extract, dissolved in methanol, was collated through a 25 cm column filled with Sephadex LH-20 [39], using methanol as the eluent. The sample was gathered in 61 different test tubes, with approximately 2 mL of sample each. 

The fractions gathered in each tube were analysed in a high-performance liquid chromatograph, HPLC (Waters Corporation, Milford, CT, USA: Pumps: 515 HPLC Pump; Injector: 717plus Autosampler; Detector: 996 Photodiode Array Detector) with a C-18 analytic column (10 × 250 mm) and using water/methanol/tetrahydrofuran (56:16:28) as a solvent at 0.75 mL/min. Twenty microliters of sample were injected. The chromatograms were set at a maximum length of 350 nm to detect the presence of flavonoids, and 250 nm for diterpenes [11,19]. The compounds were identified from the retention times and spectral characteristics [11,19]. 

The fractions with greater presence of these compounds were chromatographed again using Sephadex, until the obtained fractions showed that these compounds were separated. After repeating this procedure several times, two fractions of the exudate were finally obtained:

- Fraction 1, with the presence of diterpenes (DTP) and a dry weight of 3.031 g.
- Fraction 2, with the presence of flavonoids (FLV) and a dry weight of 0.186 g.

**Figure 4:**
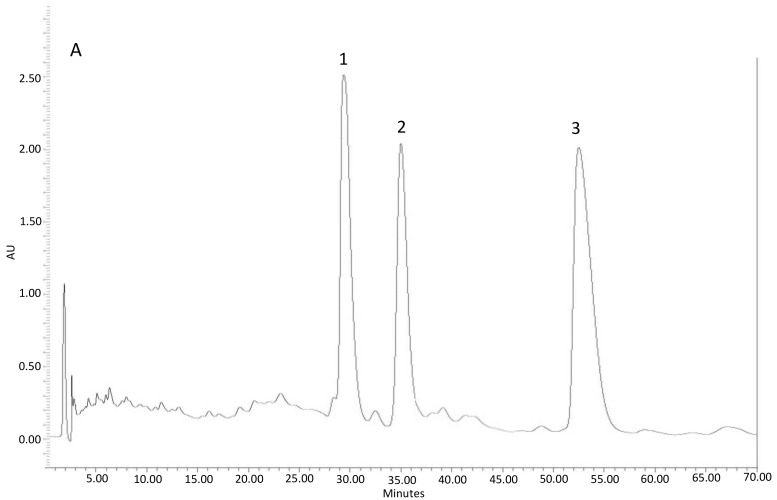

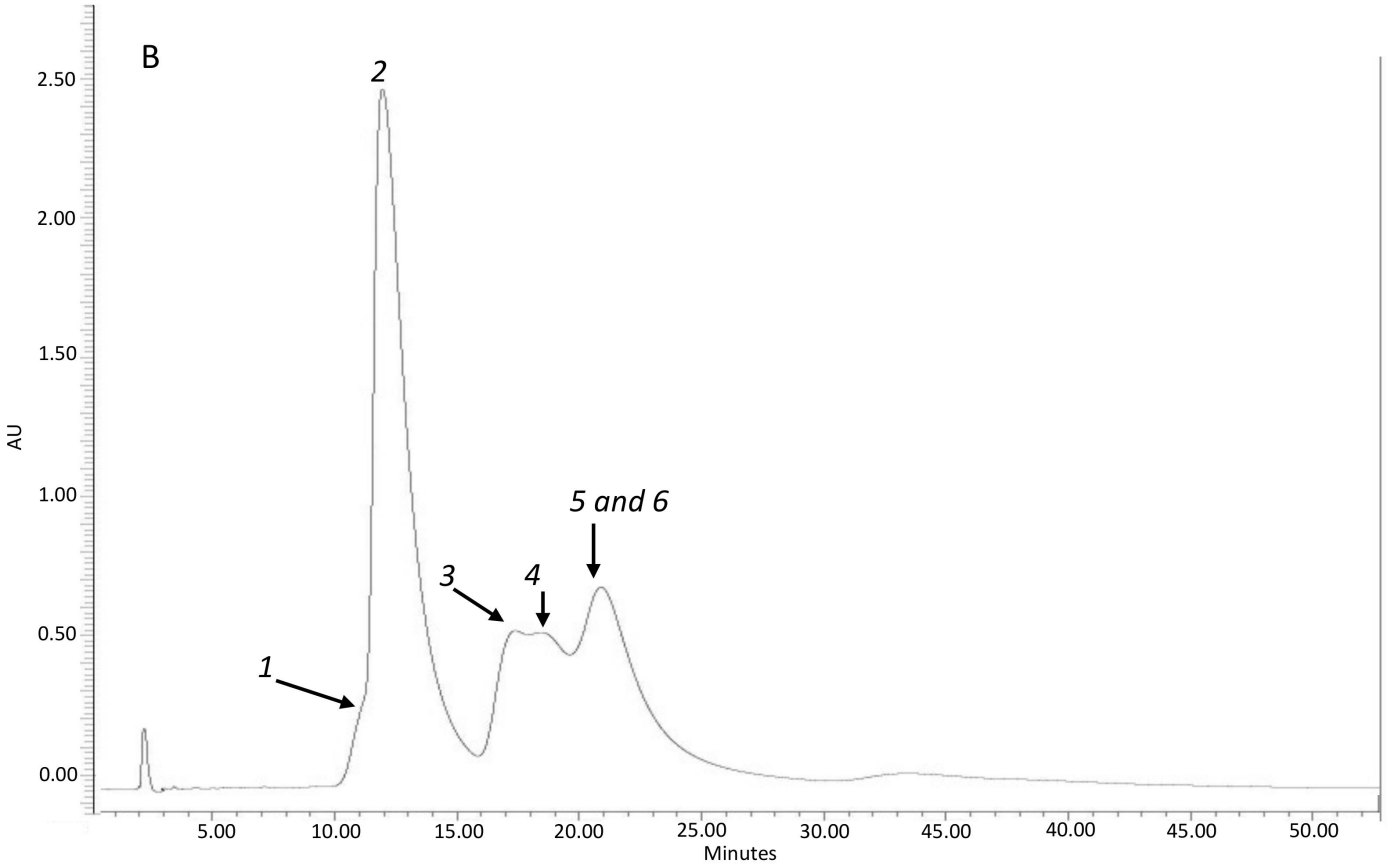
Representative HPLC-UV (diode array detector(DAD)) chromatogram of: Fraction 1 (**A**) with the presence of diterpenes: **1**: oxocativic acid, **2**: 7-oxo-8-labden-15-oic acid, **3**: -acetoxi-7-oxo-8-labden-15-oic acid; Fraction 2 (**B**) with the presence of flavonoids: **1**: Apigenin, **2**: 3-Omethylkaempherol, **3**: 4′-O-methylapigenin, **4**: 7-O-methylapigenin; **5**: 3,7 di-O-methylkaempherol.

Figure 4 shows the chromatograms of these two fractions.

Three different concentrations were tested for each of the fractions, following the concentrations tested in other studies on autotoxicity [7,33]: 0.26 g/L (concentration 1), 0.13 g/L (concentration ½) and 0.065 g/L (concentration ¼). The control concentration was Milli-Q water.

### 4.4. Germination Tests

Prior to the germination tests, the seeds of *C. ladanifer* were stimulated with dry heat, subjecting them to 100 °C for 5 min in order to interrupt their dormancy state [22].

A total of 50 seeds of *C. ladanifer* were planted in petri dishes on n° 118 Whatman paper (six replicates for each concentration of the different solutions). Initially, they were watered with 7 mL of each solution and, then, 1 mL was added every two days. The control dishes were watered with Milli-Q water. The dishes were kept in a culture chamber at constant temperature (20 °C) and a photoperiod of 16 h of light and 8 h in the dark. 

The germinated seeds were counted daily, for 15 days, considering that germination had taken place when the coating of the seed had been broken, with the subsequent emergence of the radicle. On the last day of the experiment, in each petri dish the radicle and cotyledon size were measured in 10 seedlings, which were randomly selected [40,41]. With the gathered data, the following parameters were calculated:

- *Germination percentage (%G):* (n° of germinated treated seeds/n° of germinated control seeds) × 100.
- *Germination rate (G):* This is an arithmetic measure that indicates the days needed for germination to occur. It was calculated using the formula described by [42]:
  G = [(N_1_ × G_1_) + (N_2_ × G_2_) + ….. + (N_n_×G_n_)]/G_1_ + G_2_ + ….. + G_n_ (1)
  where *G* is the germination rate, *N1*, *N2*, … *Nn* represent the number of days from the beginning of the germination test, and *G1*, *G2*, … *Gn* represent the number of seeds that germinated in day *i*.
- *Germination rate index (GRI):* This is the average number of seeds that germinate per day. It is calculated using the formula described by [43]:
  GRI = Σ (n_i_/t_i_) (2)
  where GRI is the germination rate index, *n_i_* is the number of seeds germinated in day *i* and *t_i_* is the time in days for germination in day *i*.
- *Percentage of inhibition in root size:* (root size of treated seeds/root size of control seeds) × 100.
− *Percentage of inhibition in cotyledon size:* (cotyledon size of treated seeds/cotyledon size of control seeds) × 100.

### 4.5. Statistical Analysis

To determine the existence of significant differences in the effects of the different solutions, the non-parametric U test of Mann-Whitney was used. Differences were considered significant with *p* < 0.05. The statistical analysis was performed using the SPSS software Version 22. 

## Figures and Tables

**Table 1:** Germination rate of the seeds of *C. ladanifer* watered with solutions of diterpenes and flavonoids at different concentrations (6 replicates per treatment): 1 (0.26 g/L), ½ (0.13 g/L), and ¼ (0.065 g/L). Different letters indicate significant differences between different concentrations (M-W; *p* < 0.05).

Germination Rate				
Concentration				
**Solution**	**Control**	**1**	**½**	**¼**
**Diterpenes**	4.02a	9.11b	8.10b	7.05b
**Flavonoids**	4.02a	3.60a	3.33a	3.30a

**Table 2:** Germination rate index of the seeds of *C. ladanifer* watered with solutions of diterpenes and flavonoids at different concentrations (6 replicates per treatment): 1 (0.26 g/L), ½ (0.13 g/L), and ¼ (0.065 g/L). Different letters indicate significant differences between different concentrations (M-W; *p* < 0.05).

Germination Rate Index				
Concentration				
**Solution**	**Control**	**1**	**½**	**¼**
**Diterpenes**	16.57a	5.36b	10.84c	10.00c
**Flavonoids**	16.57a	17.81a	20.16a	17.08a

## References

[1] He H.B., Wang H.B., Fang C.X., Lin Z.H., Yu Z.M., Lin W.X. (2012). Separation of allelopathy from resource competition using rice/barnyardgrass mixed-cultures. PLoS ONE.

[2] Gioria M., Osborne B.A. (2014). Resource competition in plant invasions: Emerging pattern sandresearchneeds. Front. Plant Sci..

[3] Xia R., Xiaofeng H., Zhongfeng Z., Zhiqiang Y., Hui J.X.L., Bo Q. (2015). Isolation, identification and autotoxicity effect of allelochemicals from rhizosphere soils of flue-cured tobacco. J. Agric. Food Chem..

[4] Rial C., Novaes P., Varela R.M., Molinillo J.M., Macias F.A. (2014). Phytotoxicity of cardoon (*Cynara cardunculus*) allelochemicals on standard target species and weeds. J. Agric. Food Chem..

[5] Bi X.B., Yang J.X., Gao W.W. (2010). Autotoxicity of phenolic compounds from the soil of American ginseng (*Panax quinquefolium* L.). Allelopath. J..

[6] Yang M., Zhang X., Xu Y., Mei X., Jiang B., Liao J., Zhu S. (2015). Autotoxic ginsenosides in the rhizosphere contribute to the replant failure of *Panax notoginseng*. PLoS ONE.

[7] He C.N., Gao W.W., Yang J.X., Bi W., Zhang X.S., Zhao Y.J. (2009). Identidication of autotoxic compounds from fibrou roots of *Panax quinquefolium* L. Plant Soil.

[8] Mallik A. (2003). Conifer regeneration problems in boreal and temperate forests with ericaceous understory: Role of disturbance, seedbed limitation, and keystone species change. Crit. Rev. Plant Sci..

[9] Weir T.L., Bais H.P., Vivanco J.M. (2003). Intraspecific and interspecific interactions mediated by a phytotoxin, (−)-catechin, secreted by the roots of *Centaurea maculosa* (spotted knapweed). J. Chem. Ecol..

[10] Nuñez E. (1989). Jaral Ecology of *Cistus ladanifer* L. Ph.D. Thesis.

[11] Chaves N. (1994). Variación Cualitativa y Cuantitativa de los Flavonoides del Exudado de *Cistus ladanifer* L. Como Respuesta a Diferentes Factores Ecológicos. Ph.D. Thesis.

[12] Chaves N., Escudero J.C., Gutierrez-Merino C. (1997). Role of ecological variables in the seasonal variation of flavonoid content of *Cistus ladanifer* exudate. J. Chem. Ecol..

[13] Chaves N., Escudero J.C., Dakshini K.M.N., Chester F.L. (1999). Variation of flavonoid synthesis induced by ecological factors. Principles and Practices in Plant Ecology: Allelochemicals Interactions.

[14] Sosa T., Chaves N., Alías J.C., Escudero J.C., Henao F., Gutiérrez-Merino C. (2004). Inhibition of mouth skeletal muscle relaxation by flavonoids of *Cistus ladanifer* L.: A plant defense mechanism against herbivores. J. Chem. Ecol..

[15] Chaves N., Alías J.C., Sosa T. (2016). Phytotoxicity of *Cistus ladanifer* L.: Role of allelopathy. Allelopath. J..

[16] Chaves N., Ríos J.L., Gutiérrez C., Escudero J.C., Olías J.M. (1998). Analysis of secreted flavonoids of *Cistus ladanifer* L. by high-performance liquid chromatography-particle beam mass spectrometry. J. Chrom. A.

[17] Pascual T., Urones J.G., Gonzalez M. (1977). Terpenoides monohidroxilados de la gomorresina de *Cistus ladanifer* L. An. Quim..

[18] Alías J.C. (2006). Influence of Climatic Factor Son the Synthesis and Activity of Phytotoxis Compounds Secreted by *Cistus ladanifer* L. Ph.D. Thesis.

[19] Alías J.C., Sosa T., Valares C., Escudero J.C., Chaves N. (2012). Seasonal variation of *Cistus ladanifer* L. diterpenes. Plants.

[20] Chaves N., Sosa T., Escudero J.C. (2001). Plant growth inhibiting flavonoids in exudate of *Cistus ladanifer* and in associated soils. J. Chem. Ecol..

[21] Chaves N., Sosa T., Alías J.C., Escudero J.C. (2003). Germination inhibition of herbs in *Cistus ladanifer* L. soil: Possible involvemente of allelochemicals. Allelopath. J..

[22] Pérez-García F. (1997). Germination of *Cistus ladanifer* seed in relation to parent material. Plant Ecol..

[23] Manzano P., Malo J., Peco B. (2005). Sheep gut pasaje and survival of Mediterranean shrub seeds. Seed Sci. Res..

[24] Alías J.C., Sosa T., Escudero J.C., Chaves N. (2006). Autotoxicity against germination and seedling emergente in *Cistus ladanifer* L. Plant Soil.

[25] Chaves N., Sosa T., Valares C., Alías J.C. (2015). Routes of incorporation of phytotoxic compounds of *Cistus ladanifer* L. into soil. Allelopath. J..

[26] Herranz J., Ferrandis P., Copete M.A., Duro E.M., Zalacaín A. (2006). Effect of allelopathic compounds produced by *Cistus ladanifer* on germination of 20 Mediterranean taxa. Plant Ecol..

[27] Li Z.-H., Wang Q., Ruan X., Pan C.-D., Jiang D.-A. (2010). Phenolics and plant allelopathy. Molecules.

[28] Sosa T., Alías J.C., Escudero J.C., Chaves N. (2005). Interpopulational variation in the flavonoid composition of *Cistus ladanifer* L. exudate. Biochem. Syst. Ecol..

[29] Dias A.S., Dias L.S., Pereira I.P. (2004). Activity of water extracts of *Cistus ladanifer* and *Lavandula stoechas* in soil on germination and early growth of wheat and *Phalaris minor*. Allelopath. J..

[30] Verdeguer M., Blázquez M.A., Boira H. (2012). Chemical composition and herbicidal activity of the essential oil from a *Cistus ladanifer* L. population from Spain. Nat. Prod. Lett..

[31] Kato-Noguchi H., Seki T., Shigemori H. (2010). Allelopathy and allelopathic substance in the moss *Rhynchostegium pallidifolium*. J. Plant Phys..

[32] Batish D.R., Singh H.P., Kaur S., Kohli R.K., Yadav S.S. (2008). Caffeic acid affects early growth, and morphogenetic response of hypocotyl cuttings of mung vean (*Phaseolus aureus*). J. Plant Phys..

[33] Ruan X., Li Z.-H., Wang Q., Pan C.-D., Jiang D.-A., Geoff Wang G. (2011). Autotoxicity and allelopathy of 3,4-dihydroxyacetophenone isolated from *Picea schrenkiana* needles. Molecules.

[34] Inderjit, Nilsen E. (2003). Bioassays and field studies for allelopathy in terrestrial plants: Progress and problems. Crit. Rev. Plan. Sci..

[35] Chung I.M., Miller D.A. (1995). Effect of alfalfa plant and soil extracts on germination and seedling growth. Agron. J..

[36] Charoenying P., Teerarak M., Laosinwattana C. (2010). An allelopathic substance isolated from *Zanthoxylum limonella* Alston fruit. Sci. Hort..

[37] Viard-Crétat F., Gallet C., Lefebvre M., Lavorel S. (2009). A leachate a day keeps the seedlings away: Mowing and the inhibitory effects of *Festuca paniculata* in subalpine grasslands. Ann. Bot..

[38] Bouhaouel I., Gfeller A., Fauconnier M.-L., Rezgui S., Amara H.S., Jardin P. (2015). Allelopathic and autotoxicity effects of barley (*Hordeum vulgare* L. ssp. *vulgare*) root exudates. BioControl.

[39] Vogth T.Y., Gülz P.G. (1991). Isocratic column liquid chromatographic separation of a complex mixture of epicuticular flavonoid aglycones and intracellelar flavonol glycosides from *Cistus laurifolius* L. J. Chrom..

[40] Jäderlund A., Zackrisson O., Nilsson M.C. (1996). Effects of bilberry (*Vaccinium myrtillus* L.) litter on seed germination and early seedling growth of four boreal tree species. J. Chem. Ecol..

[41] Pece M.G., Gaillard de Benítez C., Acosta M., Bruno C., Saavedra S., Buvenas O. (2010). Germinación de *Tipuana tipu* (Benth.) O. Kuntze (tipa blanca) en condiciones de laboratorio. Quebracho.

[42] Nakagawa (1999). Teste de Vigor Baseados no Desempenho das Plântulas. Vigor de Sementes: Conceitos e Testes.

[43] Maguire J.D. (1962). Speed of germination in selection and evaluation for seddling emergence and vigor. Crop Sci..

